# Combination of *In Silico* Methods in the Search for Potential CD4^+^ and CD8^+^ T Cell Epitopes in the Proteome of *Leishmania braziliensis*

**DOI:** 10.3389/fimmu.2016.00327

**Published:** 2016-08-29

**Authors:** Rafael de Freitas e Silva, Luiz Felipe Gomes Rebello Ferreira, Marcelo Zaldini Hernandes, Maria Edileuza Felinto de Brito, Beatriz Coutinho de Oliveira, Ailton Alvaro da Silva, Osvaldo Pompílio de-Melo-Neto, Antônio Mauro Rezende, Valéria Rêgo Alves Pereira

**Affiliations:** ^1^Department of Natural Sciences, Universidade de Pernambuco, Garanhuns, Pernambuco, Brazil; ^2^Department of Immunology, Fundação Oswaldo Cruz, Recife, Pernambuco, Brazil; ^3^Department of Pharmaceutical Sciences, Universidade Federal de Pernambuco, Recife, Pernambuco, Brazil; ^4^Department of Microbiology, Fundação Oswaldo Cruz, Recife, Pernambuco, Brazil

**Keywords:** neglected tropical diseases, cutaneous leishmaniasis, *Leishmania braziliensis*, vaccine development, CD4^+^ CD8^+^ T cell epitopes

## Abstract

The leishmaniases are neglected tropical diseases widespread throughout the globe, which are caused by protozoans from the genus *Leishmania* and are transmitted by infected phlebotomine flies. The development of a safe and effective vaccine against these diseases has been seen as the best alternative to control and reduce the number of cases. To support vaccine development, this work has applied an *in silico* approach to search for high potential peptide epitopes able to bind to different major histocompatibility complex Class I and Class II (MHC I and MHC II) molecules from different human populations. First, the predicted proteome of *Leishmania braziliensis* was compared and analyzed by modern linear programs to find epitopes with the capacity to trigger an immune response. This approach resulted in thousands of epitopes derived from 8,000 proteins conserved among different *Leishmania* species. Epitopes from proteins similar to those found in host species were excluded, and epitopes from proteins conserved between different *Leishmania* species and belonging to surface proteins were preferentially selected. The resulting epitopes were then clustered, to avoid redundancies, resulting in a total of 230 individual epitopes for MHC I and 2,319 for MHC II. These were used for molecular modeling and docking with MHC structures retrieved from the Protein Data Bank. Molecular docking then ranked epitopes based on their predicted binding affinity to both MHC I and II. Peptides corresponding to the top 10 ranked epitopes were synthesized and evaluated *in vitro* for their capacity to stimulate peripheral blood mononuclear cells (PBMC) from post-treated cutaneous leishmaniasis patients, with PBMC from healthy donors used as control. From the 10 peptides tested, 50% showed to be immunogenic and capable to stimulate the proliferation of lymphocytes from recovered individuals.

## Introduction

The leishmaniases constitute an important group of neglected tropical diseases (1), which affect and impact “the bottom billion” of people living in poverty by inducing disfiguration, loss of productivity, and a burden of 3.3 million disability-adjusted life years (DALY) (2–4). It is estimated that one-quarter of the world’s population, 1.7 billion people, are living in risk areas for leishmaniasis (5). Until recently, 98 countries have reported cases of leishmaniasis with 0.7–1.2 and 0.2–0.4 million cases reported annually of cutaneous leishmaniasis (CL) and visceral leishmaniasis (VL), respectively (6). The leishmaniases are caused by protozoans from the genus *Leishmania*, transmitted to humans and other mammals by phlebotomine sand fly bites (7). These diseases have multiple clinical forms and the main ones are CL, widely distributed among targeted populations and which affects the skin and mucous; VL, the more lethal form if not treated, which affects mainly the reticuloendothelial system of the liver and the spleen; and mucocutaenous leishmaniasis (MCL), which affects the mucous and has a poor prognosis. In Brazil, most of CL cases are caused by *Leishmania braziliensis*. The disease is found in different regions (8), but many patients experience spontaneous cure that is associated with IFN-γ production (9). Diverse strategies are being used to control the leishmaniasis, namely, the control of infected animals and vectors and the chemotherapy of affected individuals. These approaches, however, have a high cost and can also induce resistance in parasites and vectors (10). Therefore, a safe and effective vaccine against human leishmaniasis is urgent to address these issues.

Vaccines against *Leishmania* spp. have the major task of correctly activating the immune system to develop a protective response composed mainly by CD4^+^ and CD8^+^ T cells, producing and secreting IFN-γ. This response has been associated with disease control, macrophage activation, and parasite elimination from the host (11–13). Our previous data show that cells from patients infected with *L. braziliensis* produce high amounts of IFN-γ after stimulation with whole lysed parasite (14, 15). To initiate cellular response, dendritic cells (DCs), which are specialized antigen-presenting cells (APCs), have the unique capacity of priming naive T cells by presenting peptide antigens bound to major histocompatibility proteins (MHCs), co-stimulating and secreting cytokines, and thus mounting a T cell response against *Leishmania* spp. Some *Leishmania* species, e.g., *Leishmania amazonensis* and *Leishmania mexicana*, may fail to activate DCs, and consequently, no effective T cell response is mounted (16, 17). *L. braziliensis* is capable of activating DCs and inducing a protective immune response (18). It is estimated that each mature DC expresses 10^6^–10^7^ MHC Class II (MHC II) and 10^5^ MHC Class I (MHC I) molecules (19). The activation of CD8^+^ T cells is a result of the specific engagement of 9-mer-peptide to MHC I proteins (9-mer-p-MHC Class I), while CD4^+^ T cells are activated by 15-mer-peptides bound to MHC II (15-mer-p-MHC Class II).

There is no such thing as an ideal antigen, and the search for antigens that could generate immunogenic epitopes for a potential vaccine against *Leishmania* spp. is thus critical. In this sense, reverse vaccinology has been constantly increasing its value, and now diverse *in silico* approaches are available for the identification of potential antigens and epitopes for vaccines. Since experimental methods are difficult and time consuming, reverse vaccinology using *in silico* methods has narrowed the vast amount of molecules to be tested, increasing the odds of finding better candidates (20). In addition, many pathogen genomes and proteomes are currently available in public data banks and can be assessed regarding their potential antigen diversity and variability. Thus, sequence- and structure-based methods investigating the binding affinity of peptides to MHC I and MHC II molecules and other parameters may aid in the search for new antigens in order to support vaccine development (21). John et al. (22) have used only sequence-based methods to search for different epitopes in the predicted proteome of *Leishmania* spp. Agallou et al. (23) have recently reported the construction of a multi-epitope peptide vaccine against leishmaniasis by analyzing four known proteins from *Leishmania infantum*. In this context, we hypothesized that a combination of modern sequence and protein structure algorithms would help the search, within the whole predicted proteome from *L. braziliensis*, for potential immunogenic epitopes with high affinity for both human MHC I and MHC II. Thus, the aim of this work was to combine robust *in silico* approaches in the search for potential immunogenic T cell epitopes, based on the proteome of *L. braziliensis*, for the development of an anti-*Leishmania* vaccine.

## Results

### Linear Epitope Prediction

**Figure 1:**
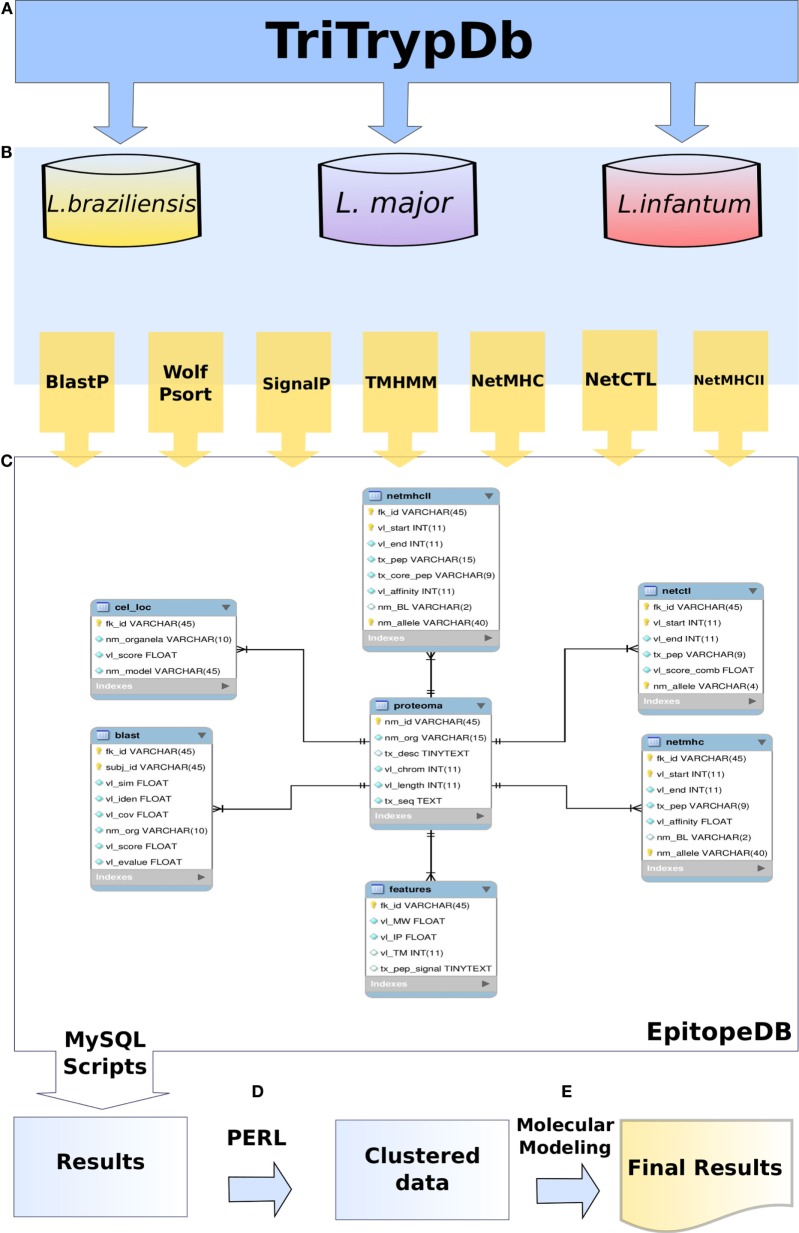
**Methodology flowchart used in this work**. TriTrypDB was used to retrieve the predicted proteome of *Leishmania* spp. **(A)**. Predicted proteome sequences were analysed by different methods **(B)**. EpitopeDB relational database was created, and managed using MySQL as database management system. Parsers and algorithms in PERL and SQL languages were developed in order to access and integrate the results **(C)**. Data was clustered **(D)**. Clustered data was used for molecular modeling **(E)**.

With the goal of developing a peptide vaccine based on *in silico* approaches, many studies have shown its feasibility, and different attempts have been carried out in order to find good epitopes capable of stimulating the immune system and its memory arm (21, 24–26), but these mainly rely on computational tools, which focus on epitope prediction. Here, we have developed an *in silico* pipeline combining the linear prediction of epitopes with a sequence of structural refinements to confirm the potential of some epitopes to bind to MHC molecules and thus stimulate the immune system. Several computational tools were applied with the goal of minimizing the number of candidate epitopes identified as well as maximizing their potential as inducers of protective immunity. The whole strategy used can be seen in the workflow described in Figure 1.

The initial epitope prediction tools used here (NETMHC and NETCTL) were selected based on two criteria, namely, predictors of MHC I and MHC II binding affinity and predictors in which their accuracy and performance applied to trypanosomatid protein sequences have already been assessed by Resende et al. (27). NETMHC and NETCTL were then used to predict MHC I-binding epitopes, and NETMHC Class II was used for a MHC II prediction. For each allele supertype, the epitopes predicted were those classified by the tools as strong binders. Simultaneously, different sets of sequence analysis were performed in order to exclude all epitopes that belonged to proteins conserved in humans and mice, so as to avoid potential autoimmune epitopes; select epitopes belonging to proteins conserved between different *Leishmania* species, potentially able to induce an immune response against multiple species; select epitopes that came from proteins, which were predicted as extracellular or secreted and having a maximum of one *trans*-membrane domain, therefore selecting epitopes from proteins easier to express and which should be generally exposed to the host immune system. The number of predicted epitopes for both MHC I and MHC II derived from these predictions, and the allele supertypes, are summarized in Table 1. If all predictions for different MHC alleles are considered, the total number of epitopes found in this stage is 657, 6,710, and 64,553, for NETMHC, NETCTL, and NETMHCII, respectively. Next, as the total epitope prediction includes some degree of redundancy, a clustering step was performed, considering the sequence similarity among the predicted epitopes, and this analysis resulted in 168 groups for MHC I and 2,138 groups for MHC II. Subsequently, each group was dismembered to reveal the exact sequence of each epitope, resulting in 230 individual epitopes for MHC I and 2,319 epitopes for MHC II.

**Table 1:** **Number of predicted epitopes by bioinformatics tools**.

Allele supertype	MHC Class I prediction	
	NETMHC	NETCTL
	
	Number of predicted epitopes	
HLA-A1	17	556
HLA-A2	181	685
HLA-A3	86	579
HLA-A24	18	444
HLA-A26	61	515
HLA-B7	20	1,620
HLA-B27	229	1,092
HLA-B44	31	466
HLA-B58	14	753

**Allele supertype**	**MHC Class II prediction**	
	
	**NETMHC Class II**	
	
	**Number of predicted epitopes**	

HLA-DPA	7,021	
HLA-DPB	1,558	
HLA-DQA	14,762	
HLA-DRB	41,212	

### Fitting Linear Epitopes into MHC Structures

**Figure 2:**
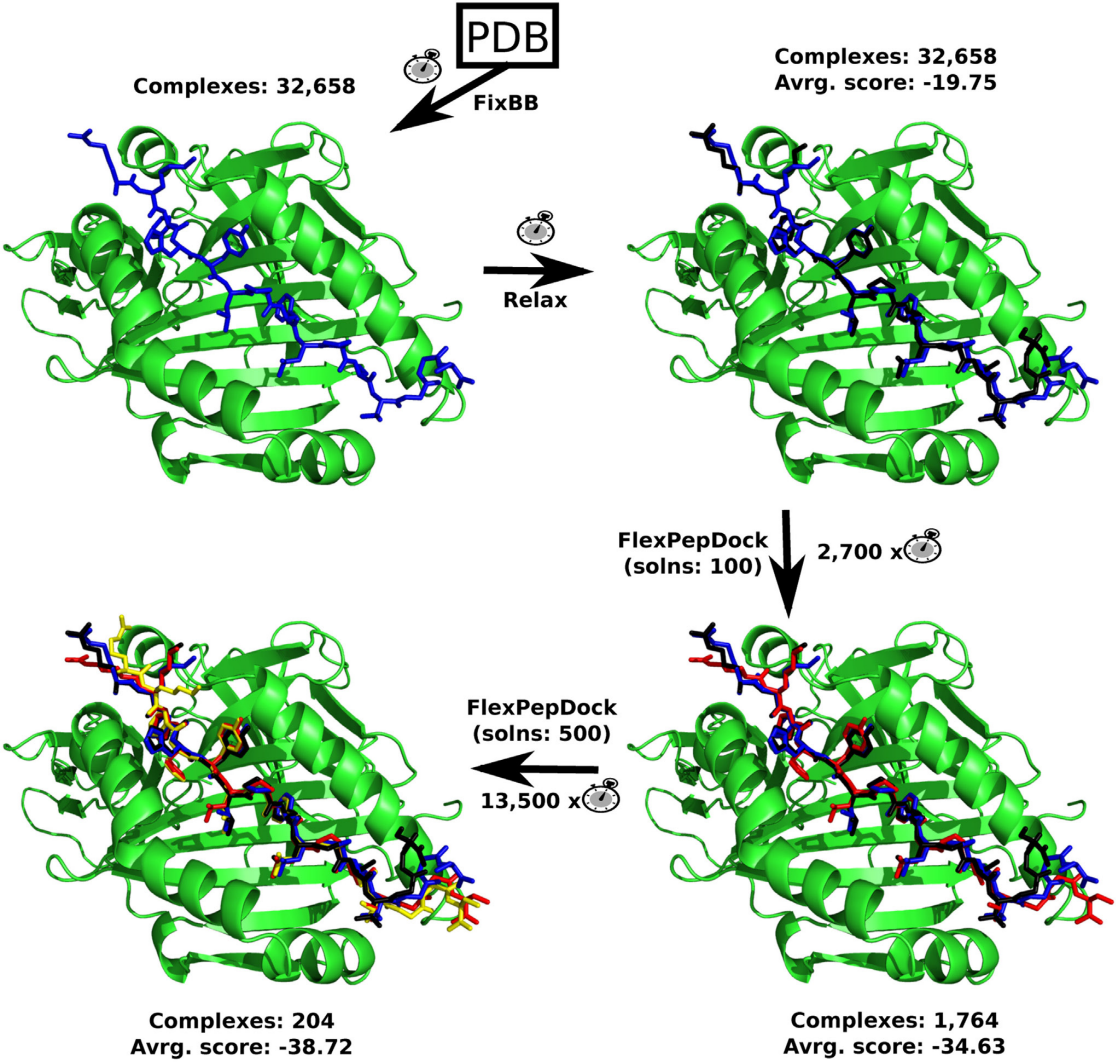
**The sequence on which the Rosetta’s protocols were used**. The total number of complexes generated and the average Interface score (Isc) of it are shown. The computational demand of each protocol compared to the FixBB protocol (clock picture), for a single complex (epitope + allele), can also be found. Every complex image consists of an example containing a MHC II allele (PDB: 3LQZ) and the epitope #1,677, after each protocol. The MHC II allele is in green, while the predicted epitope color range from blue (FixBB) to black (Relax), red (FlexPepDock with 100 solutions), and yellow (FlexPepDock with 500 solutions).

As the main objective of this work was to select predicted epitopes with a good binding affinity to a large number of MHC receptor alleles, a molecular modeling approach was used aiming to find the most *in silico* stable epitope + allele complexes. Structures from 33 different alleles of MHC I (21) and MHC II (12) were downloaded and processed as described in the Section “Materials and Methods,” prior to modeling their interaction with the individual predicted epitopes. The sequence of steps followed can be found in Figure 2. The first step consists of the replacement of the co-crystallized peptide (present at each downloaded structure) for each one of the predicted epitopes. The total number of complexes is composed of 4,830 complexes for MHC I (21 MHC I alleles times 230 predicted epitopes) and 27,828 complexes for MHC II receptor (12 MHC II alleles times 2,319 predicted epitopes). As the overall combinations of all the MHC structures and predicted epitopes achieved an impressive number of 32,658 complexes, a distributed computing strategy was adopted to process this large-scale problem in a feasible time.

With all the 32,658 complexes (receptor + epitope) generated and their respective epitopes energetically relaxed, the molecular docking could be started. This was carried out using the Rosetta’s FlexPepDock protocol. However, during the development of this molecular modeling protocol, several preliminary evaluations were made in order to find a good trade-off between precision and computational demand. First of all, it has been noticed that the use of Rosetta’s FixBB protocol to replace the co-crystallized peptide by the predicted epitopes generates typical unstable structures, with high positive Interface scores (Isc). Therefore, three complexes (predicted epitope + allele), that had the best, a regular and the worst Isc values, based solely on the structures obtained by FixBB, were selected and submitted to the Rosetta’s Relax protocol. This step was performed to verify if the Relax protocol could stabilize the selected prediction in more favorable conformations. The Relax protocol results can be found at Figure S1 in Supplementary Material. It is possible to notice an increase in affinity of the complexes (predicted epitope + MHC receptor) after the relaxing step.

### Filtering Epitope–MHC Complexes

After the analyses performed previously, there were still an impracticable number of complexes (32,658) to be used as input for Rosetta’s FlexPepDock protocol. This universe of complexes demands a computational effort that is not feasible even using a computational grid environment. Thus, a filtering strategy had to be adopted in order to select the most promising predicted epitopes to the largest possible number of MHC receptor alleles. First, knowing that epitopes containing 9 residues (MHC I) can be windows of 15-residue epitopes (MHC II), the epitopes from the Class I prediction were matched with the Class II epitopes in order to find windows of Class I and Class II epitopes. A total of 385 pairs of 15-residue epitopes and their respective 9-residue windows were found.

**Figure 3:**
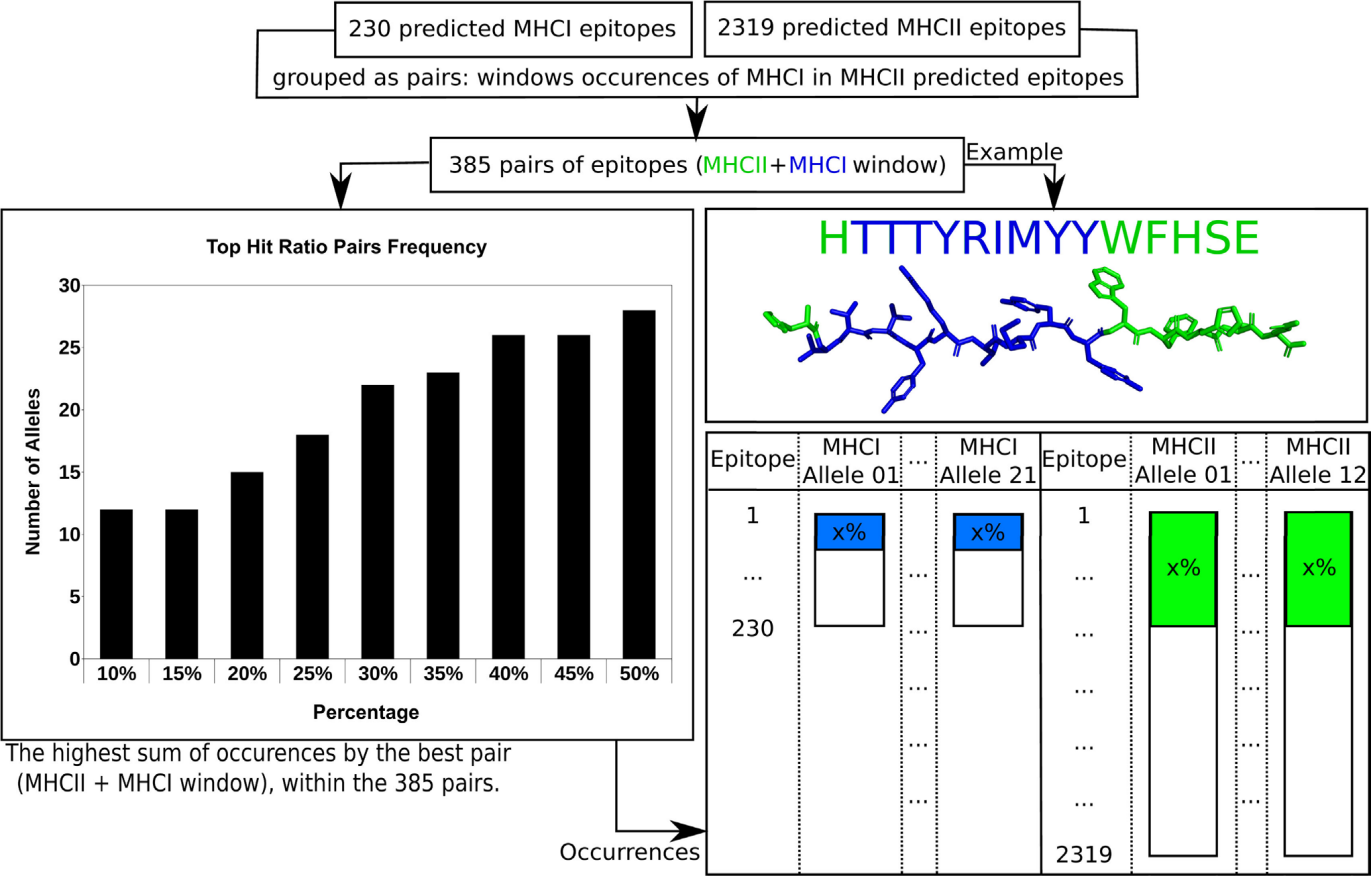
**The filtering approach used in order to reduce the number of complexes calculated at FlexPepDock step**. Details can be found in text.

The filtering strategy required the Isc scores derived from the calculation of the whole set of epitope + allele complexes, computed using the Rosetta’s Relax protocol (called rescore procedure, as detailed in the Section “Materials and Methods”). These Isc scores were used to estimate the frequency that each predicted epitope appears on the list of top 30% ranked candidates for all available MHC structures (Figure 3). If a 15-residue epitope has a good affinity for a MHC II allele, at the top 30% best scored epitopes for each target structure, and its 9-residue window also has a good affinity for a MHC I allele, this “pair” of predicted epitopes (MHC II and MHC I) might be a good candidate for better immunogenic properties. The 385 pairs of 15-residue epitopes and their respective 9-residue windows were then sorted according to the sum of their frequency within the 30% best scored epitopes. After the exclusion of repetitions, the 30% cutoff recovered 81 and 285 predicted epitopes of MHC I and MHC II, respectively, varying from 12 (at 10 and 15%) to 18 (at 25%), within the total 33 alleles (21 for MHC Class I and 12 for MHC Class II). Choosing a cutoff larger than 30% would not increase significantly the number of occurrences. Figure S2 in Supplementary Material shows the plot of percentage cutoff for the top ranked candidates, ranging from 10 to 50%, and Figure S3 in Supplementary Material presents the filtering algorithm.

Based on such filtering approach, the 100 best-ranked pairs (or 1,764 predicted epitope + allele complexes) were used at the next molecular docking step, with Rosetta’s FlexPepDock protocol, because this number of calculations required a viable computational demand, about 18-fold lower than the initial set of complexes (32,658).

### Molecular Docking

The 100 best-ranked pairs after the filtering strategy were then used at the molecular docking step with 100 docked solutions each. From this set, the top 10 pairs of predicted epitopes, with the best average Isc among the alleles, were selected for an enhanced run of Rosetta’s FlexPepDock protocol with 500 docked solutions, hence increasing the chance of finding new docking solutions with higher affinities for these 10 pairs of predicted epitopes.

**Figure 4:**
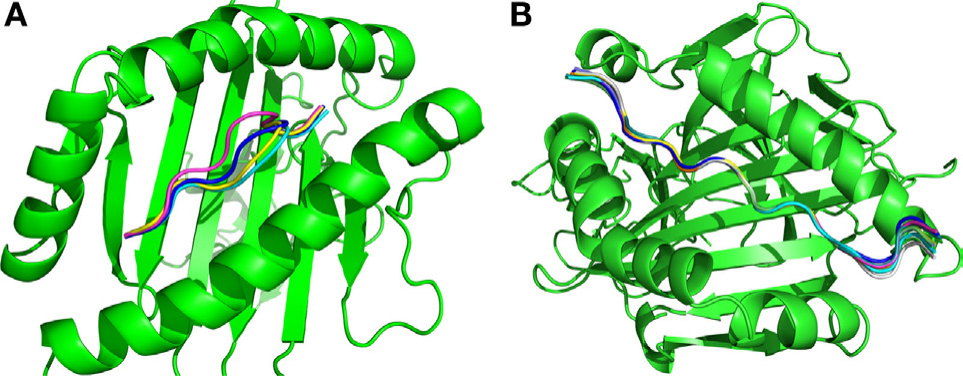
**Superimposition of the best solutions for the (A) 4 predicted MHC I and (B) 10 MHC II epitopes**.

The final results obtained through the molecular docking of these 10 pairs of predicted epitopes defined a total of 4 unique 9-residue epitopes predicted for MHC I and 10 unique 15-residue epitopes predicted for MHC II. Figure 4 shows the superposition of the best-docked solutions derived from the alleles with the highest binding affinity (lowest Isc) to their respective MHC targets [alleles with Protein Data Bank (PDB) IDs 4NQV and 3LQZ for MHC I and MHC II, respectively]. One can see that the best docking solutions are quite similar in position, displaying a homogeneous result. The way these solutions are positioned are consistent with the known binding mechanism for these MHCs, as each predicted epitope bound to the correct key anchor residues localized within the binding groove of its corresponding MHC target (28). The MHC I (Figure 4A) predicted epitopes were anchored by residues located at the epitope extremities, while the MHC II predicted epitopes (Figure 4B) were anchored by residues positioned in the middle. The final selected 14 predicted epitopes are those that displayed the best binding affinity among the 2,549 candidate epitopes (230 targeting MHC I + 2,319 targeting MHC II).

### Epitope–MHC Interaction Features

**Figure 5:**
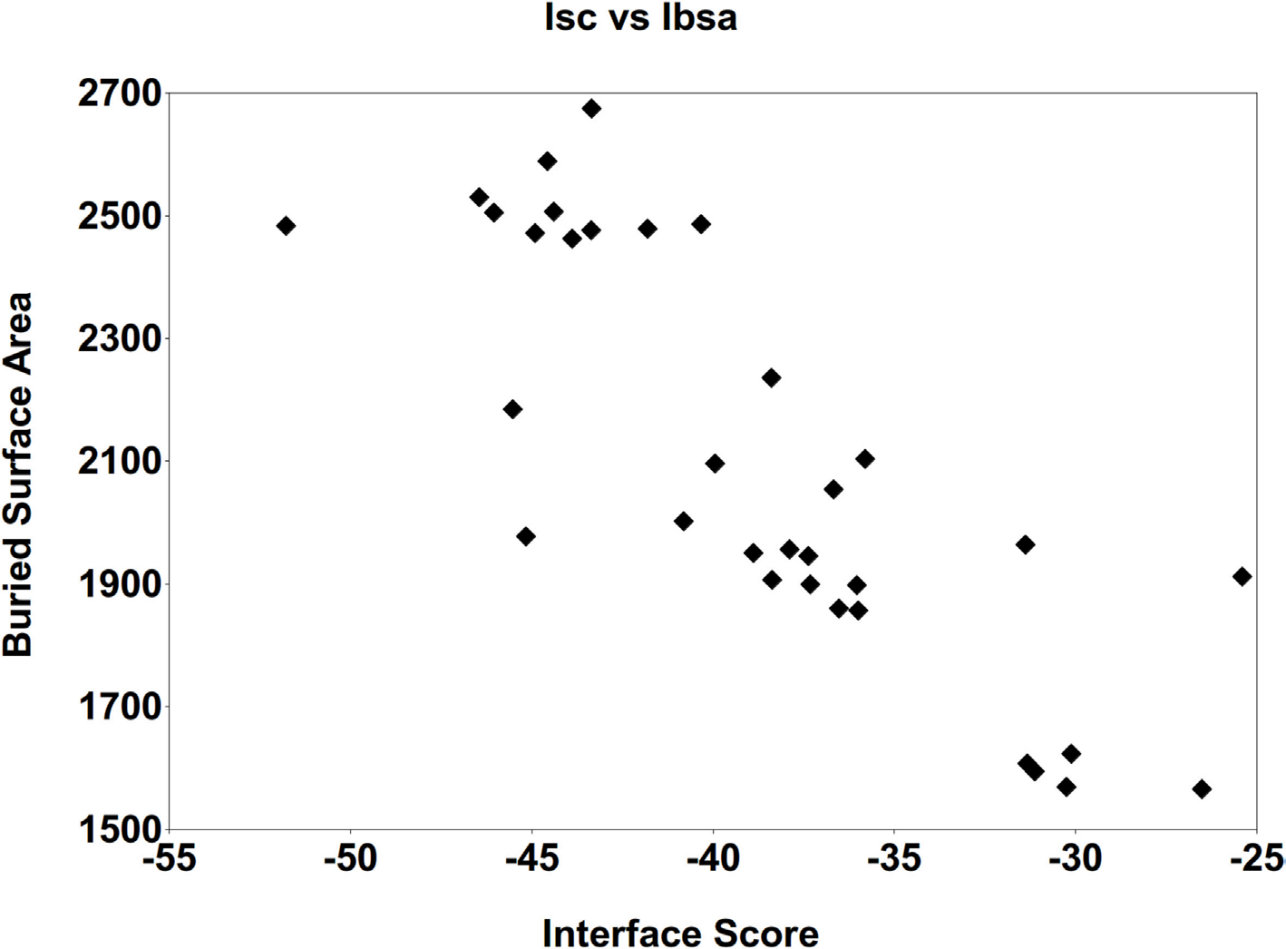
**Correlation between the average Interface-buried surface area (Ibsa, in square angstroms) and the average Interface score (Isc)**. This was performed for all the complexes generated with the 33 MHC receptors, after the FlexPepDock protocol (with 500 solutions).

Detailed analyses were then conducted in order to identify important intermolecular aspects responsible for the affinity of these final predicted epitopes to the MHC targets, as follows. First, the correlation between the Interface-buried surface area (Ibsa), commonly used to measure the size of the macromolecule interface (29), and the Isc was evaluated. Figure 5 presents the average Ibsa and Isc values for all the complexes (epitope + allele) formed by each one of the 33 MHC structures. A strong correlation between Ibsa and Isc was seen, with bigger Ibsa values being accompanied by lower (more stable) values for Isc. This was to be expected, since when the contact area between the ligand (predicted epitope) and the receptor (allele structure) is larger, there are more intermolecular interactions between them stabilizing the complex, with lower (more negative) Isc values. Thus, an increase on Ibsa contributes in a favorable way to the binding affinity of the complexes, by typically lowering the Isc.

**Figure 6:**
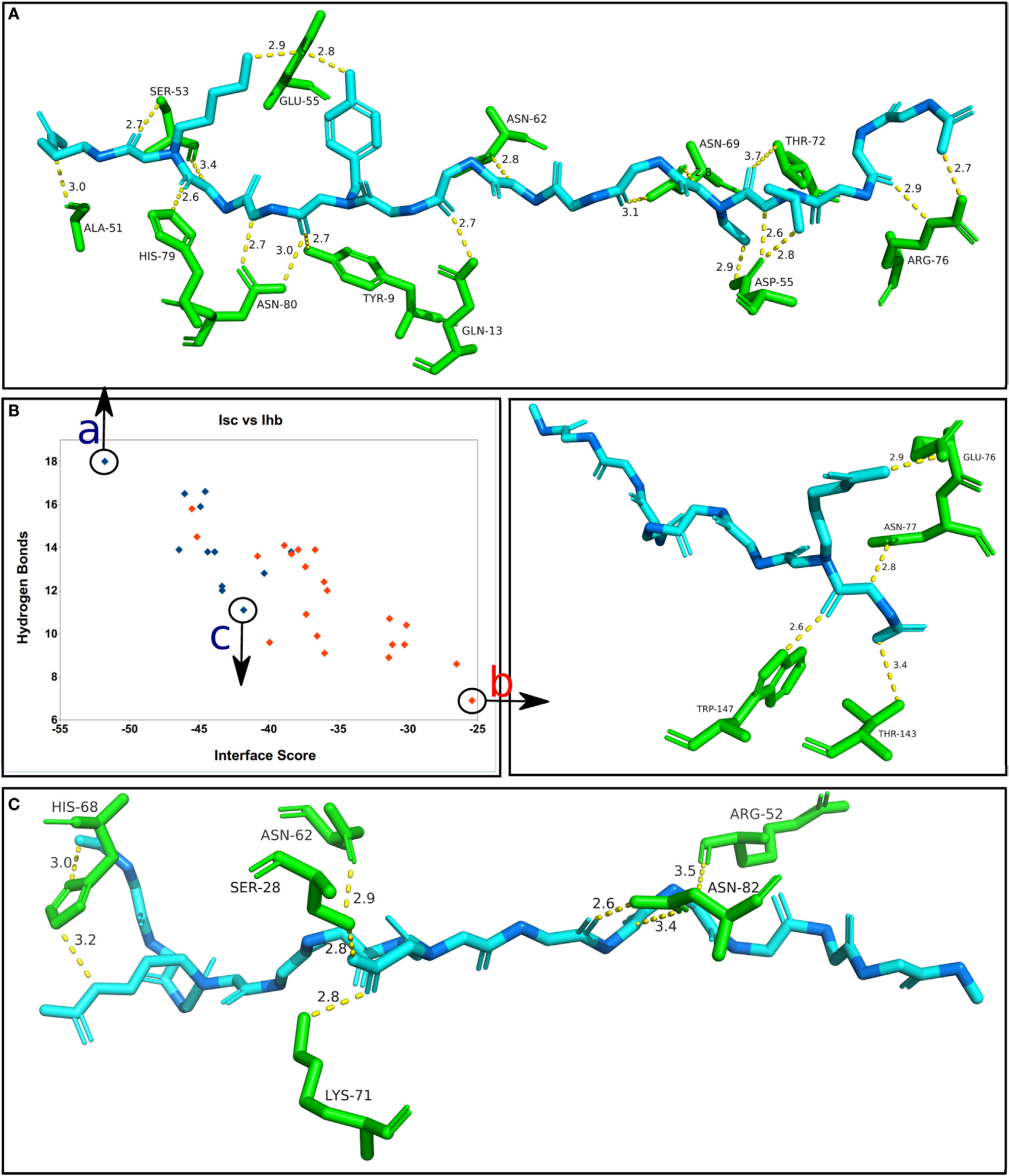
**Correlation between the average Interface score (Isc) and the average Interface hydrogen bonds (Ihb), for the complexes obtained by the FlexPepDock protocol with 500 solutions (FPD500)**. The blue dots correspond to the MHC II alleles (total of 12), while the red dots correspond to the MHC I alleles (total of 21). **(A)** The best docking solution of the final set of results, showing the predicted epitope that formed the highest number of hydrogen bonds and generated the lowest (most stable) Isc. **(B)** The worst overall solution within the final results, having a low number of hydrogen bonds and the highest (less stable) Isc value. **(C)** One of the worst solutions for a MHC II complex, having a reduced affinity between the allele and the predicted epitope. Only the side chain groups with hydrogen bonds are shown. The epitope’s alpha-carbons are highlighted in dark blue.

In a similar analysis to the one presented in Figure 5, Figure 6 shows the correlation between the average Interface hydrogen bonds (Ihb) and the average Isc values for all the complexes (apitope + allele) formed by each one of the 33 MHC structures. As more hydrogen bonds are formed between the receptor and the predicted epitope, the Isc value is lower, i.e., the complex has a higher binding affinity, emphasizing the fact that the peptides bind to the MHC alleles (particularly MHC II) *via* an “extensive hydrogen bond network” (28). To further investigate this correlation, three complexes included in the final results (FPD500) were selected and analyzed (Figure 6). It is important to notice that the larger Class II epitopes (with 15 residues) have a greater natural probability to form hydrogen bonds, because of the higher number of residues (two-thirds more). It is also important to emphasize that the average Isc, highlighted in Figure 2 as “Avrg. score,” can be observed as progressively more negative with each successive step of the methodology applied, meaning more stable epitope + allele complexes identified during the *in silico* procedures.

### Epitope Tracing

Next, the identity of the 10 highest ranking epitopes was investigated, and they were seen to derive from a total of 4 *L. braziliensis* proteins. The first of these, a conserved hypothetical protein, is encoded by a CDS localized to chromosome 34 and encompasses at least 4 potential epitopes. Interestingly, the protein segment encompassing the 4 15-mer epitopes is 19 aa in linear length. Therefore, the four potential epitopes with high affinity for MHC I and MHC II molecules are found within this particular segment, with minor differences between each of these four epitopes, but all producing high scores when analyzed by the approaches described above. Another conserved hypothetical protein located on chromosome 1 encodes at least three epitopes. Here, the three epitopes were found in a window 18 aa in length. The third protein, found in chromosome 14, encodes two 15-mer epitopes located in a window of 16 aa. Finally, the last and largest protein, also hypothetical, encodes only one 15-mer epitope. All the genes encoding for those proteins are syntenic with other genes from trypanosomatids. This indicates that these proteins have evolved in the same genetic loci from diverse trypanosomatids, and the potential epitopes may induce cross-protection against other pathogenic species from the same family.

### Validation of Peptide Epitopes

**Figure 7:**
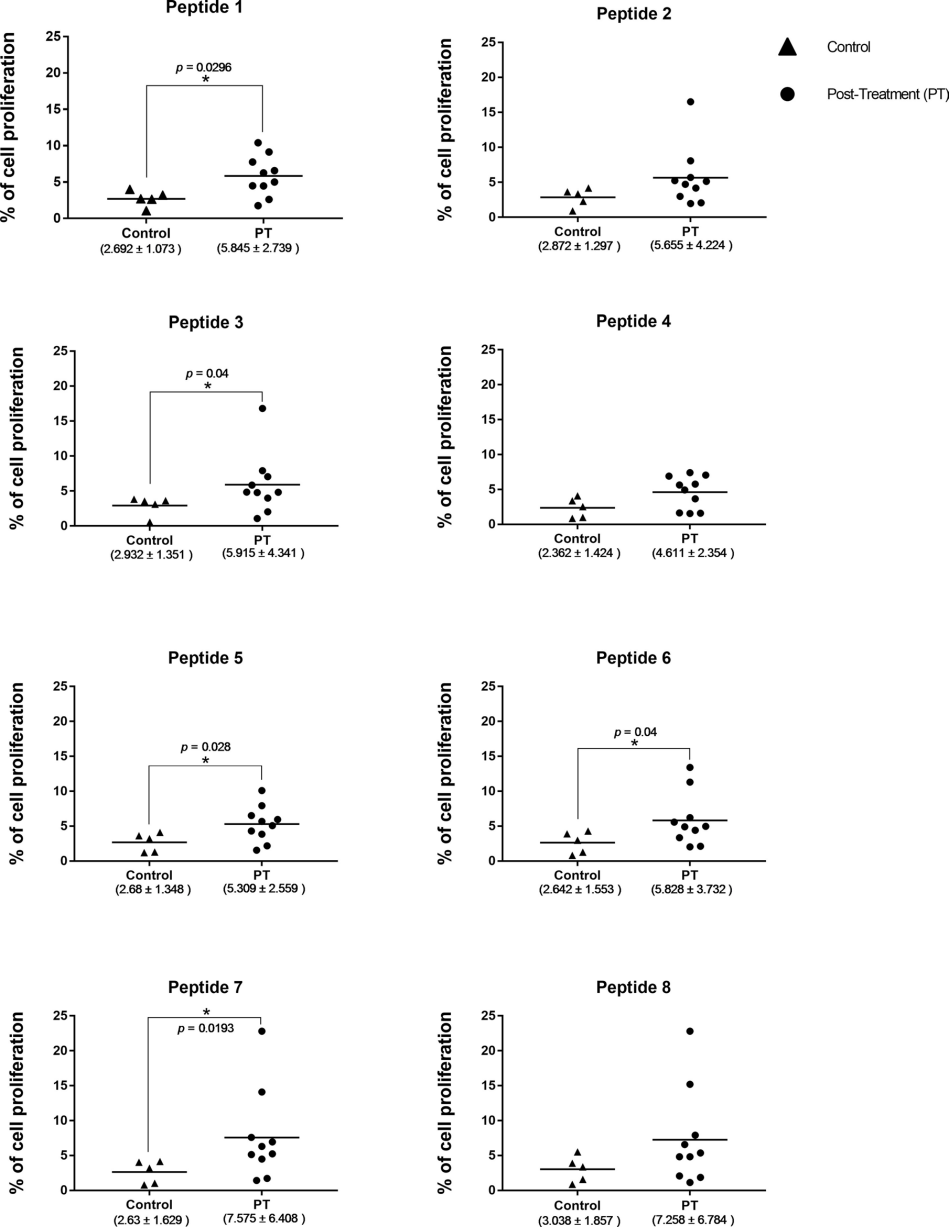

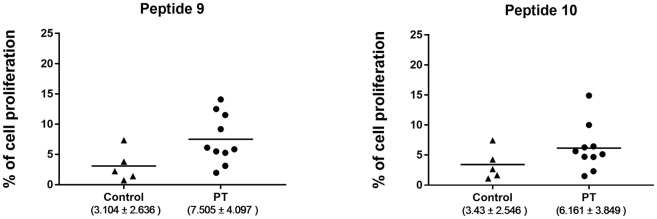
**Comparison of the proliferation data from PBMC derived from the cutaneous leishmaniasis post treatment (PT) patients with the PBMC proliferation data from the control group, in response to different peptides**. The asterisk indicates significant differences (*p* < 0.05) between patients and control group. The horizontal bars represent the mean values for each group. The median percentage levels and the corresponding SD for each group tested are described below.

In order to have an experimental validation of the results derived from the bioinformatic approach, peptides corresponding to the 10 highest ranking 15-mer epitopes described above were commercially synthesized. These were then used for an evaluation of their ability to induce proliferation of peripheral blood mononuclear cells (PBMC) derived from human patients cured after treatment. These PBMC were capable of proliferating when stimulated with total antigen from *L. braziliensis* (7.6 ± 6.1), and non-stimulated cells had minimal levels of proliferation (data not shown). The results from the assays carried out with the synthetic peptides are presented as the mean percentage of proliferation, with its SD, calculated with the data from the proliferation of PBMC derived from 10 patients, when exposed to the individual peptides. Peptides 2, 4, 8, 9, and 10 were capable of stimulating proliferation of many of the PBMC derived from the afflicted patients. However, no significant difference was observed when the mean proliferation values obtained for these peptides with the PBMC from the 10 patients was compared with the mean values derived from the data with PBMC from the control group, consisting of five healthy volunteers. In contrast, significant statistical differences were observed for the mean PBMC proliferation results between samples from treated patients and control volunteers for the assays carried out with peptides 1, 3, 5, 6, and 7 (Figure 7). These five peptides are derived from three of the four *L. braziliensis* proteins described above, confirming that the positive response is not associated with one specific protein. The possibility that the improved results from some peptides might also be associated with a stronger response from a reduced number of individuals was also investigated. The PBMC from two patients did respond better to a greater number of peptides, four in all, but these include peptides included among both groups described above. In all, the PBMC proliferation data highlight the potential of the computational approaches used for epitope selection and indicate that some of the synthetic peptides tested would be recognized by the immune system to mount a protective immune response.

## Discussion

So far, different approaches have tried to address the lack of an anti-*Leishmania* vaccine capable of being effectively used against the leishmaniasis. However, most of the vaccines under development have failed in very early assays, due to factors such as poor antigen response, absence of good animal models, and lack of standardization (30). An ideal vaccine should also be capable of stimulating a promiscuous response against different *Leishmania* species, but generally only one species has been considered at a time. Moreover, only a few papers have described predictions of T cell epitopes from *Leishmania* spp. proteomes (31–35). Most of these have focused on epitopes, which bind to MHC I, with a focus on CD8^+^ T cell response and not considering the induction of a response mediated by CD4^+^ T cells (31, 32, 35, 36). CD8^+^ T cells have a major role in protecting against CL, but evidence has been provided that they may also exacerbate and compromise the disease outcome (37). In this context, we have searched for natural epitopes that would stimulate both CD4^+^ and CD8^+^ T cells, in order to establish a more balanced response that could favor the prevention of disease progression. However, development of CD4^+^ T cell predicted epitopes is still a challenge, since we do not fully understand the epitope motifs that bind to the MHC II groove, most of the peptides may not be naturally processed by APCs, and the positional alignment is critical for the prediction (38, 39). To overcome these issues, the epitopes predicted here were tested through different criteria in order to define a final set of peptides. One of these criteria was the capacity to bind with high affinity to both MHC I and MHC II, as demonstrated by the high scores of the complexes and the elevated number of hydrogen bonds, for example. Thus, it is expected that, during natural processing by APCs, these peptides could bind either to MHC I or MHC II or be able to bind both molecules. We hypothesized that the differences observed in the cell proliferation are a consequence of the number of T cells present in the peripheral blood of recovered patients. In addition, these peptides may differ in how they are presented and activate T cells since they have different linear sequences.

In terms of target MHC molecules, this work has crucially focused in simulating peptide epitopes with different allele supertypes expressed by different human populations. Allele selection considered their distribution across the globe and other important parameters, such as the promiscuity of the selected peptides to which they bind and PDB crystallized structures. To do so, most of the work was based on human MHC alleles and in order to reduce bias due to MHC multiplicity, allele supertypes were used (40–42). Supertypes share specific residues at some anchor positions, thus, they are capable of binding to overlapping groups of peptides (43). It is important to consider the human MHC molecules, since the purpose of the work is to develop a vaccine that would be applied to humans. Previous works have already helped the search for potential epitopes that could bind to murine MHC, since it is the most used experimental model for preclinical assays, testing potential vaccines against leishmaniasis (23, 36). Most of the *in silico* developed peptides based on the murine model, however, may fail to translate good results to humans, due to the huge differences between human and mouse immune responses. All the computational efforts performed here were therefore dedicated to simulate peptides with human MHC alleles.

One of the major problems of bioinformatics these days is the huge amount of data generated. Linear epitope predictions using predicted proteomes provide a large number of potential epitopes, which cannot possibly be tested. Therefore, this large number must be reduced into a feasible number of epitopes, which can be experimentally tested for their immunogenicity. Thus, the option here to solve this issue was for molecular modeling protocols, mainly through the application of molecular docking approaches, adding another layer of strength to the data. Here, it has been shown that the best-ranked peptide epitopes are clearly those that establish the highest number of molecular interactions (like hydrogen bonds and hydrophobic contacts, for example) with the chemical groups in the MHC groove. Nevertheless, the *in silico* methods employed and some decisions taken during the process may have imposed some limitations. One of these is that, the tools available to predict linear epitopes use learning machine techniques, which have a huge dependency on training dataset. However, those tools are not currently trained with experimental data from trypanosomatids, such as *L. braziliensis* (27). Additionally, other factors have also guided the decisions, such as the exclusion of proteins with more than one transmembrane helix that might be difficult for further expression, and exclusion of proteins conserved but with high similarity with human sequences, which is not desired for a vaccine. Moreover, one limitation related to structural approach is the limited diversity of HLA structure alleles available on the PDB bank. Here, just 21 and 12 alleles of MHCI and MHCII, respectively, were tested. This aspect could affect the performance of the method used here.

Based on the results presented so far, it seems that the proposed combination of approaches is consistent enough to be applied in cases of reverse vaccinology, when there is a large quantity of candidate epitopes to be tested. The strategy of distributed computing (computational grid) alongside the filtering algorithms has turned an unpractical problem, into a feasible task, done in weeks. Moreover, in the context of leishmaniasis, the results of this research identified peptide epitopes with high potential to stimulate the immune system to develop a protective response.

## Materials and Methods

### Linear Epitope Prediction

#### Proteome Retrieval and Conservancy

The available proteomes (from *L. braziliensis, Leishmania major*, and *L. infantum*) were downloaded from TriTrypDB (44) and used to perform, in parallel, different bioinformatics analyses. Only protein sequences from *L. braziliensis* with more than 60% conservancy with other *Leishmania* species verified through BLAST protein alignment were considered for epitope analysis. This parameter was taken into account since an ideal vaccine should be capable to induce protection in individuals against as many species as possible.

#### MHC Class I and MHC Class II Prediction Tools and Binding Affinity Prediction

NetMHC and NetCTL tools were used for a MHC I predictions, while MHC II predictions were made using the NetMHCII tool for the most prevalent allele supertypes (45–47). Both NetMHC and NetCTL are epitope predictors; however, NETMHC just predicts the epitope, while NETCLC also considers other predictions, such as the transport efficiency prediction mediated by the transporter associated with antigen processing (TAP) protein and the C-terminal proteasomal cleavage prediction. The cutoff score defined to select peptides with high affinity for those tools was ≥1 in order to maximize the number of true positive predictions.

#### Similarity and Biological Features of Protein Candidates

In order to exclude protein candidates with high degree of similarity with proteins of humans and mice, the BLAST sequence alignment tool (48) was used to compare parasite protein sequences against host protein sequences. Proteins with degree of similarity equal or higher than 40% with human or mice proteins were excluded from the next steps. Moreover, transmembrane helix and subcellular localization predictions were performed using the TMHMM tool (49) and WoLF PSORT (50), respectively.

All the data obtained after running the methods described above were deposited in a relational database, which is managed using MySQL as a database management system (DBMS). Parsers and algorithms in PERL and SQL languages were developed in order to access and integrate the results deposited in the databank.

#### Clusterization

An in-house algorithm based on BLAST alignment results was developed in order to group the data with high similarity. A threshold of 60% of identity and 100% of coverage between any two epitopes were used in order to cluster them in the same group. In addition, group selection was performed based on selective criteria: peptide epitopes with high affinity predicted for at least three different allele of either MHC Class I or MHC Class II, or peptide epitopes derived from at least three different proteins.

### Molecular Modeling Approach

#### Preparing MHC Structures from PDB

The structures from the 33 different alleles of MHC I (21 PDB structures: 2HJL, 3C9N, 3HCV, 3KPP, 3L3D, 3RL1, 3VCL, 3VFS, 3 × 11, 4F7M, 4G8G, 4HWZ, 4JQX, 4MJ5, 4MJI, 4NQV, 4O2C, 4QRR, 4QRU, 4WU5, and 4XXC) and MHC II (12 PDB structures: 1A6A, 1BX2, 1H15, 1S9V, 1UVQ, 1YMM, 2NNA, 2Q6W, 3C5J, 3LQZ, 3PL6, and 3WEX) were downloaded from RCSB PDB (51). These structures were then prepared by removing the water molecules, ligands, and duplicated residues or alleles. Furthermore, using the PyMol (52) software in build mode, the co-crystallized small peptide chain, for each structure, was modified to have the same length as the predicted peptides used in this work (9 residues for MHC I and 15 residues for Class II). In addition, each non-canonical amino acid found in the co-crystallized peptide was manually mutated to alanine.

An in-house developed software named GriDoMol was then used to prepare and submit into a computational grid environment all the *in silico* procedures required to combine the MHC structures to the predicted epitopes and to compile the results obtained at each step into formatted datasheets. This computational grid environment was assembled, in our laboratory, by using eight computers, each containing 2× Intel Xeon quadcore (total of eight cores per computer) chipset and 16 GB RAM memory.

#### Producing MHC–Epitope Complexes

The sequence of steps on which the Rosetta framework (53) protocols were used can be found in Figure 2. For the replacement of the co-crystallized peptides for each one of the predicted epitopes, the Rosetta’s FixBB protocol, available within the Rosetta framework, was used. However, the Rosetta’s FixBB protocol does not move the backbone atoms, and thus the replacement of the co-crystallized peptide for a new one may produce unstable final conformations. Therefore, the Rosetta’s Relax protocol was used, right after the FixBB protocol, in order to energetically stabilize each one of the new epitopes. Moreover, all the MHC receptor’s residues have been locked unmovable to prevent conformational changes on the receptor side, while only the epitope’s residues were allowed to move and rotate toward a more stable conformation in the chemical neighborhood.

In order to quantify the binding affinity between receptors and predicted epitopes, at this particular step, the score function named Isc was chosen from the Rosetta’s FlexPepDock protocol (54), which is the sum over the energetic contributions of the interface residues on both the receptor and the predicted epitope. Hence, all the 32,658 structures obtained by the application of the Rosetta’s Relax protocol were only rescored using the Rosetta’s FlexPepDock protocol, in order to obtain the Isc values, but keeping the same structure obtained by Relax protocol (i.e., without any change on the atomic coordinates). In other words, the rescore procedure just recomputed the energy (with a better scoring function) at the same geometry, without perturbing the chemical system.

#### Scoring Potential Epitopes through Molecular Docking

While each run of the FixBB or Relax protocols takes a few seconds to generate the result, each run of the FlexPepDock protocol, using the default setup (100 docked solutions obtained as result), takes hours to complete. Thus, a filtering strategy had to be adopted in order to select the most promising predicted epitopes, based on their Isc scores (obtained by rescoring, as mentioned above) with receptors and the frequency of affinity observed along different MHC receptor structures.

The Rosetta’s FlexPepDock protocol, using the refinement approach, was applied to perform the molecular docking, allowing the full flexibility for the predicted epitope and the side chain flexibility for the residues at the receptor’s interface. This procedure searched for the predicted epitopes with the best binding affinities for MHC’s alleles. For each predicted epitope, the best docking solutions were selected according to the Isc. At the end, the predicted epitopes were ranked by the average Isc of their solutions along the MHC’s alleles, granting an overall view of each epitope’s average affinity. From the list of 100 pairs previously described, the first 10 pairs of predicted epitopes with the best average Isc among the alleles have been selected for an enhanced run of Rosetta’s FlexPepDock protocol, increasing the number of generated structures from 100 to 500. The computational cost of such calculation (500 docked solutions) was about 5 times greater than the default 100 docked solutions.

### Validation of Peptide Epitopes

#### Synthetic Peptides and Storage

Peptides corresponding to the top 10 ranked 15-mer peptides were synthesized (Genome Biotechnology, Brazil). Linear peptides were purified through a high performance liquid chromatography (HPLC) approach with a final purity greater than 95%. All the synthetic peptides were individually resuspended in DMSO and stored at −80°C until use.

#### Sampling and Isolation of Peripheral Blood Mononuclear Cells

All individuals included in this research signed a written informed consent before blood collection, following recommendations of the Ethics Committee from the Centro de Pesquisas Aggeu Magalhães (CPqAM-FIOCRUZ, Project: 522.964). From each individual, a total of 30 mL of peripheral blood was collected by venipuncture in sodium-heparin tubes (Vacuette, USA). The blood was diluted (1:1 v/v) with phosphate-buffered saline (PBS, pH 7.2) and deposited onto the Ficoll–Paque PLUS density (1.077 g/mL) gradient (GE Healthcare, USA) and centrifuged. Subsequently, the PBMC layer was individually removed and washed twice with PBS.

#### CFDA-SE Labeling and Cell Culture

About 4 × 10^6^ cells were resuspended in 1 mL of PBS containing 2 μM of carboxyfluorescein diacetate succinimidyl ester (CFDA-SE, Invitrogen, USA) and incubated at 37°C for 10 min. The CFDA-SE concentration was previously titrated in order to prevent inhibition of cell proliferation or cell death. After incubation, cell labeling was quenched with 1 mL of ice-cold (4°C) RPMI 1,640 containing 2 mM of l-glutamine, 50 mg/L of gentamicin sulfate, and 2 mg/L of amphotericin B, supplemented with 10% fetal bovine serum (both Cultilab reagents, Brazil). The cells were pelleted and washed with PBS followed by resuspension in 1 mL of RPMI 1,640 supplemented at a density of 2 × 10^6^ cells/mL. The PBMC were plated in 96-well U bottom plates (BD Falcon) at a density of 2 × 10^5^ cells/well with 20 μg/mL of each peptide. These cells were then incubated at 37°C with 5% CO_2_ for 96 h. For each patient or control volunteer tested, non-stimulated and phytohemagglutinin (PHA)-stimulated cells were evaluated as intra-experimental controls.

#### Flow Cytometry Analysis

The analyses were performed on a FACScalibur flow cytometer (Becton Dickinson Company, USA) equipped with an argon laser (wavelength 488 nm). Fluorescence of 20,000 lymphocyte gated events, based on scatter parameters of size and granulosity, was acquired. The data were analyzed and treated with FlowJo v10.1 (Tree Star Inc., USA). Non-stimulated cells were used during the analysis for setting quadrant parameters and to set the basal level of lymphocyte proliferation. For the statistical analysis, the data were analyzed with non-parametric Mann–Whitney *U*-test. Differences were considered statistically significant when *p* < 0.05.

## Author Contributions

Conceived and designed the methods: RS, MH, AR, and VP. Performed the *in silico* approaches: RS, LF, MH, and AR. Performed the epitope validation: RS, VP, MB, BO, and AS. Analyzed the data: RS, LF, MH, Od-M-N, AR, VP, MB, BO, and AS. Wrote the paper: RS, LF, MH, Od-M-N, AR, and VP.

## Conflict of Interest Statement

The authors declare that the research was conducted in the absence of any commercial or financial relationships that could be construed as a potential conflict of interest.
