# Supplementary material for: Combination of In Silico Methods in the Search for Potential CD4+ and CD8+ T Cell Epitopes in the Proteome of Leishmania braziliensis
Source: Front Immunol. 2016 Aug 29;7:327. doi: 10.3389/fimmu.2016.00327 (PMC5002431; doi:10.3389/fimmu.2016.00327)
Supplement: Supplementary file 1 [file Data_Sheet_1.DOCX]

Supplementary Material

**Combination of *in silico* methods in the search for potential CD4^+^ and CD8^+^ T cell epitopes in the proteome of *Leishmania braziliensis***

Rafael de Freitas e Silva^1,2^, Luiz Felipe Gomes Rebello Ferreira^3^, Marcelo Zaldini Hernandes^3^, Maria Edileuza Felinto de Brito^2^, Beatriz Coutinho de Oliveira^2^, Ailton Alvaro da Silva^2^, Osvaldo Pompílio de-Melo-Neto^4^, Antônio Mauro Rezende^4*¶^,

Valéria Rêgo Alves Pereira^2*¶^

^1^Department of Natural Sciences, Universidade de Pernambuco, Garanhuns, Pernambuco, Brazil

^2^Department of Immunology, Fundação Oswaldo Cruz, Recife, Pernambuco, Brazil

^3^Department of Pharmaceutical Sciences, Universidade Federal de Pernambuco, Recife, Pernambuco, Brazil

^4^Department of Microbiology, Fundação Oswaldo Cruz, Recife, Pernambuco, Brazil

**^*^Co-corresponding authors**

E-mails: antonio.rezende@cpqam.fiocruz.br, valeria@cpqam.fiocruz.br


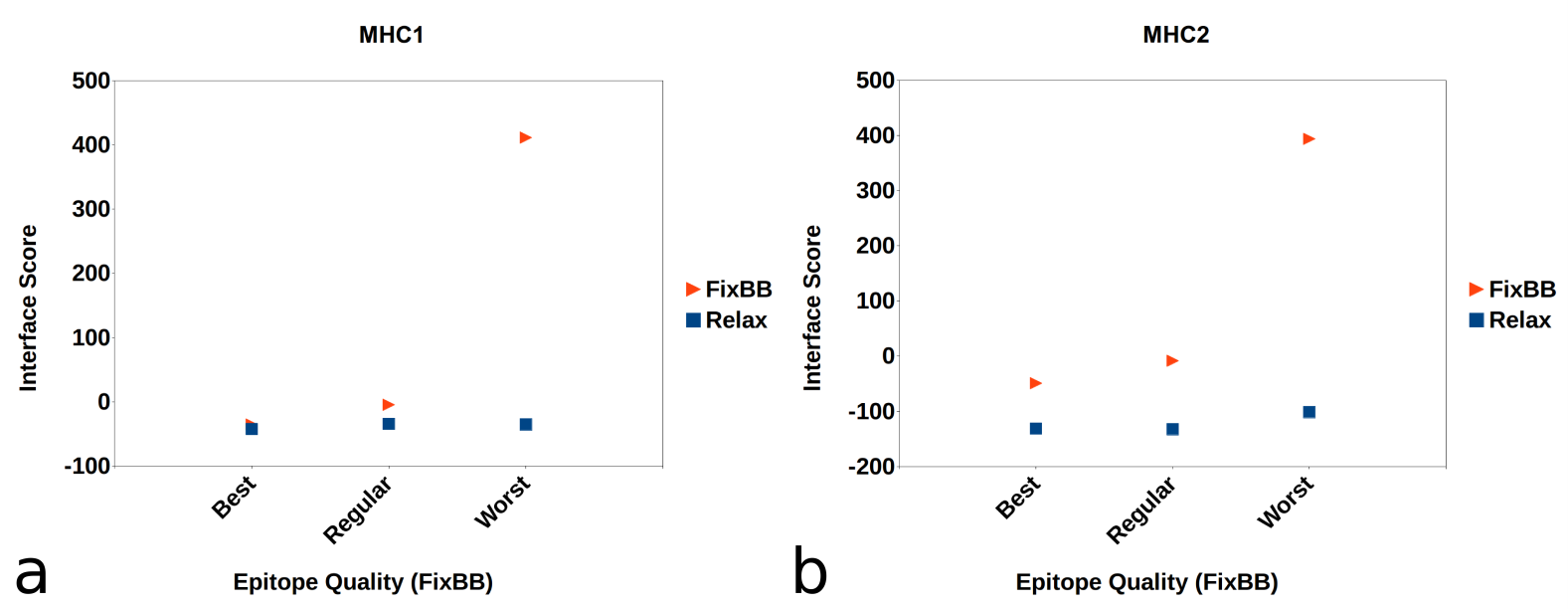


**Supplementary Figure 1.** Comparison of the Isc values found for the conformations obtained with FixBB (red triangles) and Relax (blue squares) protocols (Rosetta), for both MHCI (**a**) and MHCII (**b**). The predicted epitopes selected to this evaluation are classified as "best", "regular" and "worst" (see text for details).


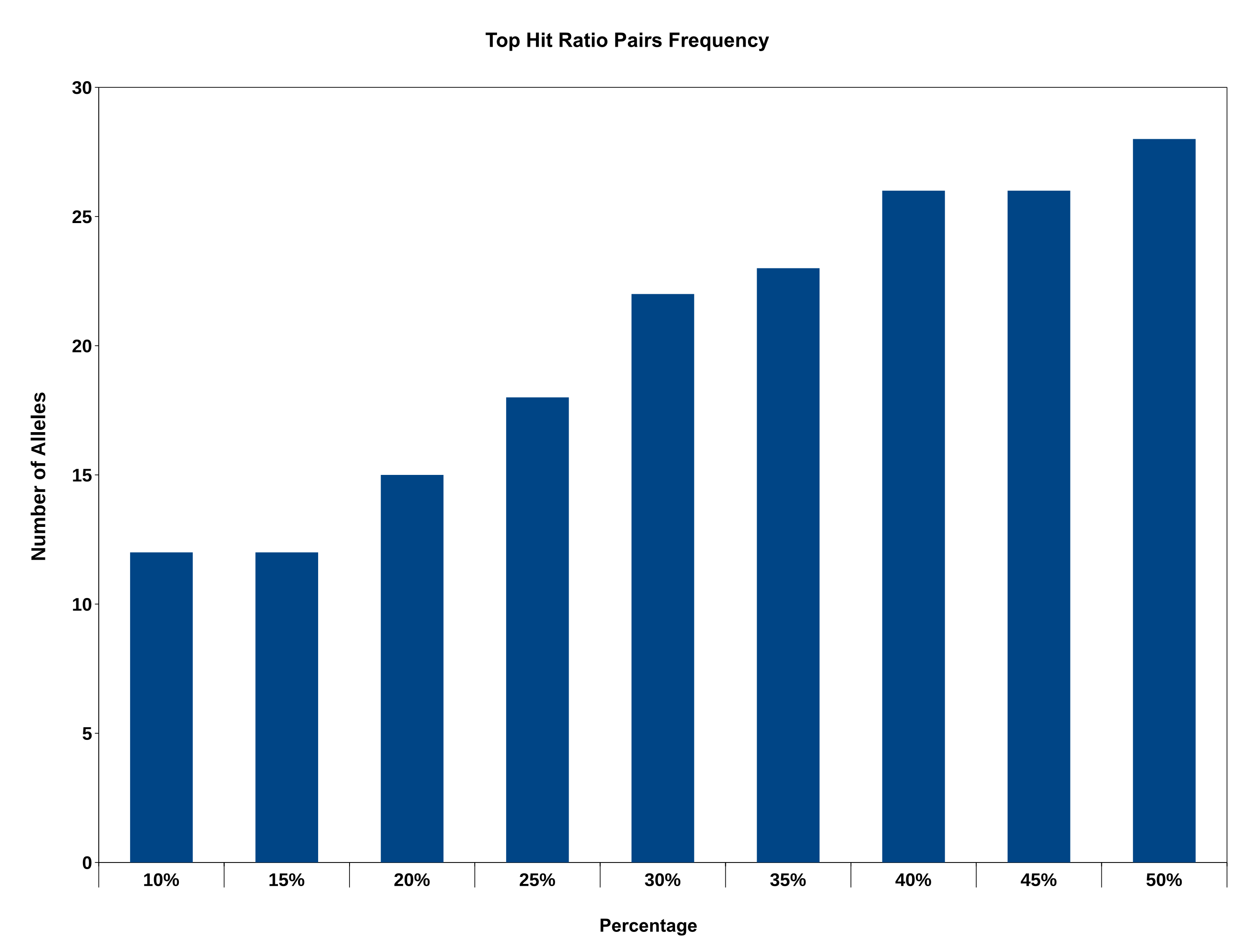


**Supplementary Figure 2.** The highest sum of occurrences of the top epitope pair (MHC II + MHC I window) found for each cutoff (%).





**Supplementary Figure 3.** Filtering algorithm, using the example of 30% cutoff.
